# The Role of the Skin in Interoception: A Neglected Organ?

**DOI:** 10.1177/17456916221094509

**Published:** 2022-08-15

**Authors:** Laura Crucianelli, H. Henrik Ehrsson

**Affiliations:** Department of Neuroscience, Karolinska Institutet

**Keywords:** interoception, skin, thermosensation, insula, body awareness

## Abstract

In the past 2 decades, interoception has received increasing attention in the fields of psychology and cognitive science, as well as neuroscience and physiology. A plethora of studies adopted the perception of cardiac signals as a proxy for interoception. However, recent findings have cast doubt on the methodological and intrinsic validity of the tasks used thus far. Therefore, there is an ongoing effort to improve the existing cardiac interoceptive tasks and to identify novel channels to target the perception of the physiological state of the body. Amid such scientific abundancy, one could question whether the field has been partially neglecting one of our widest organs in terms of dimensions and functions: the skin. According to some views grounded on anatomical and physiological evidence, skin-mediated signals such as affective touch, pain, and temperature have been redefined as interoceptive. However, there is no agreement in this regard. Here, we discuss some of the anatomical, physiological, and experimental arguments supporting the scientific study of interoception by means of skin-mediated signals. We argue that more attention should be paid to the skin as a sensory organ that monitors the bodily physiological state and further propose thermosensation as a particularly attractive model of skin-mediated interoception.

A traditional classification for the perception of body-related stimuli relies on a distinction between exteroception (i.e., signals originating from outside the body), proprioception (i.e., signals about the position and movements of our limbs and body parts; Proske & Gandevia, 2012), and interoception (i.e., signals that provide information about the internal status of the body; for a review, see Ceunen et al., 2016). The interplay between exteroceptive, proprioceptive, and interoceptive signals is important to update to maintain a coherent representation of our own body and for bodily awareness (Crucianelli et al., 2018; Dijkerman, 2015; Ehrsson, 2020). For decades psychological and neuroscience research has mainly focused on the perception of exteroceptive stimuli, such as visual and auditory signals and discriminative touch (Bermúdez et al., 1995), and in the field of body-representation research most work has concentrated on proprioception and the integration of proprioceptive and exteroceptive signals (e.g., Collins et al., 2005; Graziano, 1999). More recently, there has been a substantial increase in attention and research on interoception (see also Khalsa et al., 2018).

There are several definitions of interoception (see Table 1), and there is still no consensus on the one that should be adopted (for recent reviews, see Chen et al., 2021; Quigley et al., 2021). Thus, there is a tendency to embrace the definition that suits the scientific approach or method used in each study. In its original definition, interoception was conceptualized as the body-to-brain axis of sensations concerning the state of the visceral body and its organs (Cameron, 2001; Sherrington, 1948; see Table 1), thus involving signals originating from within the body (e.g., cardiac, respiratory, and digestive functions). However, physiological and anatomical observations led to redefining and extending interoception to encompass information about the physiological condition of the entire body, including signals originating from many tissues of the body, such as the skin (e.g., temperature, itch, pleasure from gentle touch, pain), and conveyed by specialized afferent pathways (Craig, 2002; Ceunen et al., 2016). In particular, the ideas highlighted in this article are in line with the more inclusive definition provided by Craig, according to which interoception represents the perception of the physiological condition of the entire body at any given time (Craig, 2002; see Table 1). As such, the skin represents not only the boundaries of our body but also plays a fundamental role in homeostatic regulation by monitoring externally and internally generated signals about the body’s physiological state of relevance for such self-regulation and, ultimately, survival (Björnsdotter et al., 2010; Burleson & Quigley, 2021; Craig, 2003, 2008; Crucianelli et al., 2022; Ehrsson et al., 2007; von Mohr and Fotopoulou, 2018).

**Table 1.** Summary of the Most Used Definitions of Interoception

Reference	Definition
Sherrington (1906)	The sensory nerve receptors that react to stimuli originating within the body
Sherrington (1948)	Body-to-brain axis of sensations concerning the state of the visceral body and its internal organs
Ádám (1998)	Processing of information that is picked up by sensory receptors innervating the internal organs and transmitted by ascending pathways of the autonomic nervous system
Cameron (2001)	Visceral sensory nervous system impulses connecting body to brain to behavior and thought, with or without awareness
Craig (2002)	The sense of the physiological condition of the body at any given time
Damasio (2010)	The sensing of the organism’s interior
Critchley et al. (2004)	The sensing of the internal state of the body
Dworkin (2007)	Sensory visceral receptors that monitor the internal state of the body
Barrett & Simmons (2015)	The perception and integration of autonomic, hormonal, visceral, and immunological homeostatic signals that collectively describe the physiological state of the body
Ceunen et al. (2016)	A multimodal integration not restricted to any sensory channel or mere sensations but also relying on learned associations, memories, and emotions and integrating these in the total experience, which is the subjective representation of the body state
Khalsa et al. (2018)	The overall process of how the nervous system senses, integrates, stores, and represents information about the state of the inner body
Oxford English Dictionary (n.d.)	Any form of sensation arising from stimulation of interoceptors and conveying information about the state of the internal organs and tissues, blood pressure, and the fluid, salt, and sugar levels in the blood

The perception of temperature, pain, and gentle caress-like touch (which now is referred to as “affective touch”) has traditionally been classified as submodalities of somatosensation (Sherrington, 1948) and thus often conceptualized as part of an exteroceptive modality that provides information about external objects and external events occurring on the skin. For example, when we explore objects with the digits, tactile and thermosensory impressions are seamlessly combined so that we can experience both the shape and roughness of the object together with its thermal properties; thus, a smooth spherical metal object feels cooler than the same-shaped object made of wood, for example (e.g., Carnahan et al., 2010). However, although these modalities are the results of stimulation on the body surface, thermosensation, affective touch, and cutaneous pain also carry information about the physiological state of the skin and the body in line with the previously mentioned redefinition of these as interoceptive submodalities (Craig, 2003, 2008) based on their affective, functional, physiological, and anatomical characteristics (Cabanac et al., 1972; Craig, 2002; Mower, 1976). Thus, this conceptualization emphasizes that these signals provide information about one’s own body (i.e., it is *you* that feels cool or warm, pain, or the pleasure of an affective caress) to highlight the affective-emotional dimensions of these sensory experiences. A critical argument for including signals from the skin in the concept of interoception comes from neuroanatomical considerations (Craig, 2009, 2010). Noxious, thermal, and affective-touch information that is signaled by special classes of receptors in the skin reach the brain via different anatomical pathways through the spinal cord and thalamus than tactile and proprioceptive information (see further below). These signals target a different cortical area, the posterior insular cortex, which is crucial for interoception and processes visceral information. However, there is no consensus on whether certain skin signals should be defined as interoceptive.

Putting the issue of definitions aside, there is perhaps more agreement on the main function of interoception, which is subserving homeostatic regulation (i.e., the continuous neurobiological process that maintains a relative stability in the physiological condition of the body despite internal and external changes; Billman, 2020; Cannon, 1929; Craig, 2008; Quigley et al., 2021) by supporting allostasis (i.e., the process of regulating peripheral systems in the body; Kleckner et al., 2017). Interoception is related to the autonomic nervous system and the generation of bodily (affective) feelings, informing the organism about its bodily needs (Craig, 2008, 2009; Damasio, 2010; Seth, 2013). Therefore, the impact of interoception is thought to extend beyond homeostatic regulation and also relates to the experience of emotions and the awareness of ourselves as feeling entities at any given time (Craig, 2009; Critchley et al., 2004; Damasio, 1994; Zaki et al., 2012). Specifically, it has been proposed that the integration between interoceptive signals and exteroceptive information lies at the core of bodily awareness and self-consciousness (e.g., Allen & Tsakiris, 2018; Park & Blanke, 2019; Salvato et al., 2020; Simmons et al., 2013).

This article aims to discuss some of the anatomical, physiological, and experimental arguments supporting the scientific study of interoception by means of skin-mediated signals. First, we consider classic tasks to probe interoception through cardiac awareness and argue that the analysis of skin-based interoceptive signals provides a complementary and deeper understanding of interoception as a multifaceted construct. We then also pay particular attention to thermosensation, which has been understudied in this regard, and propose that this sensory modality makes for a potential good model of skin-mediated interoception and review ongoing methodological advances in this direction.

## The Problematic Assessment of Interoception

The ability to perceive interoceptive signals has traditionally been quantified by asking participants to focus on their own heartbeats without touching their body but just by feeling the sensation of their heart beating (Schandry, 1981). In classic heartbeat counting tasks, participants are instructed to count their heartbeats during specific time windows (Dale & Anderson, 1978; Schandry, 1981); an interoceptive accuracy index is then calculated using a formula that compares the numbers of actual and reported heartbeats. Given its relatively simple implementation and the quick procedure, this task became the main method used to quantify individual abilities in interoceptive accuracy (Ring & Brener, 2018). However, this task has been criticized because it is not clear whether participants are counting their own heartbeats or rather keeping track of time and/or using previous knowledge to provide their best guess. Alternative methods to measure cardiac interoception are heartbeat detection or discrimination tasks, in which participants are asked to judge whether exteroceptive stimuli (e.g., auditory or visual cues) are presented in synch or out of synch with their own heartbeats (e.g., Katkin et al., 1983; Whitehead et al., 1977). Interestingly, the performances on these two types of tasks are unrelated, suggesting that they might assess different aspects of the perception of cardiac signals, raising questions regarding how to best register accuracy (Desmedt et al., 2018; Ring & Brener, 2018). Other issues with such methods include evidence that the performance on heartbeat counting or detection tasks seems to be influenced by other factors such as prior knowledge, heart rates, beliefs, practice, and even experimental instructions (e.g., Ring & Brener, 1996; Ring et al., 2015; Ross & Brener, 1981; Whitehead & Drescher, 1980; for an extensive debate on the issues related to heartbeat tasks, see Ainley et al., 2020; Corneille et al., 2020; Zamariola et al., 2018; Zimprich et al., 2020). In addition, from a physiological point of view, the heartbeat signal itself can be problematic because it represents a multimodal, rather “noisy” signal given the concurrent vascular and muscle contractions that give rise to a cascade of other bodily signals (e.g., activation of tactile mechanoreceptors and volume of blood ejected during each heartbeat; Azzalini et al., 2019; Knapp-Kline et al., 2021). Thus, it is challenging to know whether participants are feeling the heartbeat signal per se or whether they are using other bodily strategies to complete heartbeat detection or counting tasks (e.g., changes in respiration, tensing muscles, feeling pulsations in the fingertips; Murphy et al., 2019; Ross & Brener, 1981; Whitehead & Drescher, 1980). Furthermore, cardiovascular functions offer only one limited aspect of the broad palette of interoceptive signals. To overcome such limitations, the interoceptive field has witnessed a common effort to develop novel methods to quantify interoception, either by finding better ways to target the perception of cardiac signals (e.g., Larsson et al., 2021; Legrand et al., 2021; Plans et al., 2021) or by focusing on other organs that provide interoceptive signals (for a recent debate, see Ainley et al., 2020; Corneille et al., 2020; Zamariola et al., 2018; Zimprich et al., 2020).

The maintenance of homeostasis is a sophisticated mechanism and does not rely solely on one basic function. Indeed, interoception extends beyond cardiac signals and includes other signals originating from inside the body. Along this line, a few studies have attempted to investigate interoceptive abilities by focusing on other modalities, such as gastric or stomach functions (e.g., Azzalini et al., 2019; Herbert et al., 2012; Whitehead & Drescher, 1980), respiratory or breathing tasks (e.g., Faull et al., 2019; Garfinkel et al., 2016), bladder functions (Griffiths, 2015; Ketai et al., 2016), and thermal, nociceptive, and C-tactile (CT) signals originating from the skin, which is the topic of the present work (Craig, 2002, 2009; for more details, see below).

The question of whether interoception should be considered a unitary concept or a set of relatively independent submodules is an important one, both conceptually and from empirical perspectives. Although this is an area of ongoing research, some recent studies have found that interoception might be better conceptualized as a modular construct with relatively independent processing in parallel streams (Crucianelli et al., 2022; see also Ferentzi et al., 2018; Garfinkel et al., 2016). For example, we recently investigated the relationships between cardiac interoception and several skin-mediated interoceptive modalities (i.e., pain, affective touch, and thermosensation in two tasks) and found that they are relatively independent (Crucianelli et al., 2022). Thus, it is becoming increasing clear that to achieve a deep understanding of the concept of interoception, it should be quantified using a “battery of interoceptive tests,” and attention should be paid to all channels because each comes with specificities that uniquely contribute to the full picture of interoception.

## Probing Interoception via the Skin: Evidence From Affective Touch and Cutaneous Pain

Probing interoception via external cutaneous stimuli can provide a more precise and controlled sensory signal compared with internal stimulation (e.g., Björnsdotter et al., 2010; Craig, 2002; Crucianelli et al., 2018, 2021; Fotopoulou & Tsakiris, 2017; Quigley et al., 2021). The skin, given its very nature, is a sensory organ extensively and directly exposed not only to the inside of the body but also to the external environment. Thus, one of the reasons why skin signals might have been overlooked so far is the fact that they provide both interoceptive and exteroceptive sensory information, making it difficult to disentangle the two. Nevertheless, carefully designed and controlled experiments can allow us to manipulate only one component (i.e., the interoceptive one of interest) while keeping the other constant or absent (i.e., the exteroceptive one). Given these premises, we argue that it is time to recognize the interoceptive nature of skin-mediated signals in addition to the widely studied exteroceptive facet of touch.

The increasing focus on the study of affective touch and cutaneous pain has been partially motivated by the discovery of CT afferents, a specialized group of skin afferents that has been found mainly on the hairy skin of the body (for evidence in humans, see Vallbo et al., 1999) and has been proposed as one of the key afferent systems for affective touch (Löken et al., 2009; Morrison et al., 2010). In humans, CT afferents respond more vigorously to slow, caress-like touch, provided at a temperature typical of human skin (Ackerley et al., 2014; Löken et al., 2009; Vallbo et al., 1999; Wessberg et al., 2003); this specific type of tactile stimuli is more likely to be observed during spontaneous physical social interactions (Croy et al., 2016; Morrison, 2016c; Morrison et al., 2010). Further support comes from neuroimaging studies that have shown that CT signals are processed in key brain regions associated with interoception such as the insula and cingulate cortices (for reviews, see Björnsdotter et al., 2010; Morrison, 2016a, 2016b; see further below). The characteristics and role of the CT system in affiliative behaviors, affective touch, social bonding, and the communication of emotions has been widely described and discussed and fit with the view that CT processing and the associated subjective pleasant-touch experiences should be considered an interoceptive submodality (for reviews, see Kirsch et al., 2018; Löken et al., 2009; Morrison et al., 2010; Olausson et al., 2002; Walker & McGlone, 2013).

Likewise, pain can also be conceptualized as an interoceptive feeling and motivation (Craig, 2003). Pain has historically been seen both as a sensation and an emotion. The sensory dimension—nociception—is related to the activation of nociceptors and supports spatial localization and intensity encoding of the stimulus. The motivational-affective dimension—the subjective pain—arises centrally by the further processing of nociceptive signals and integration of other sources of information (Basbaum & Jessell, 2013). This latter motivational-affective dimension is involved in coding its valence (e.g., unpleasantness) and motivational relevance and has a more complex relationship to the original peripheral nociceptive signal. Thus, similar to affective touch, in which one can distinguish between CT processing resulting from the gentle mechanical stimulation of hairy skin and the resulting subjective pleasantness experience of affective touch, nociceptive processing and pain can also be distinguished in terms of sensory processing and later affective-emotional dimensions (e.g., Auvray et al., 2010; Hofbauer et al., 2001; Kulkarni et al., 2005; Rainville et al., 1997). As in the case of hunger or thirst, pain represents a strong drive for action that includes but it is not limited to changes in behavior (e.g., withdrawing a body part), cognitive processes (e.g., trying to focus the attention on something else in the case of sustained pain), as well as social support (e.g., asking for help; Krahé et al., 2013). In line with this interoceptive-homeostatic view of pain, Morrison et al. (2013) proposed a predictive regulation and action model of acute pain processing, according to which the nervous system is organized to anticipate potential pain and to provide the motivation to take action to reduce the risk of tissue damage.

The feeling of cutaneous pleasure or pain is a common experience to all healthy human beings to various extents, but it is also subjective, as just described. This affective experience is rather the result of an elaborate and complex integration of various peripheral (i.e., activation of mechanoreceptors and nociceptors), multisensory (e.g., visual information), and contextual (e.g., social) cues and cognitive and emotional processes that provides us with a rewarding, relaxing, and calming experience in the case of affective touch (Pawling et al., 2017) or modulates a perceived unpleasantness and distressing emotion, as in the case of pain (Farrar, 2010). Thus, cutaneous pleasure and pain share more characteristics with interoceptive rather than exteroceptive modalities given their homeostatic and affective nature (Craig, 2003). As we discuss in the next section, we propose that thermosensation can be conceptualized in a similar way, arguing that it can be useful to distinguish between *thermosensory processing* and the sensory-discriminatory aspects of thermal stimulation on the one hand and the subjective affective feelings the processing of these signals also lead to, such as *thermal comfort* and *discomfort*, on the other.

## Thermosensation as an Interoceptive Modality

Human beings would not be able to survive longer than a few hours if they could not monitor or regulate their own temperature (Sherwood & Huber, 2010). The regulation of body temperature (*thermoregulation*) is one of the most vital concerns for many homeothermic animals, including humans (for a review, see Craig, 2002; Tansey & Johnson, 2015). Both breathing and thermoregulation contribute to the maintenance of homeostasis. Although we have an organ just for the regulation of oxygen needs (i.e., lungs), there is not just one organ responsible for thermoregulation. Thus, the brain and the body are capable of activating almost immediate regulatory mechanisms against undesirable challenges to core body temperature (Davies et al., 2012; Filingeri et al., 2017; Proffitt, 2006). Involuntary physiological reactions may be involved, such as shivering or sweating, and these responses are activated at an early stage. Furthermore, voluntary temperature regulation takes place almost constantly (e.g., by changing clothing or the temperature of the room), driven by thermal stimuli that are perceived at the periphery, integrated at the central level, and lead to actions or reactions. Let us discuss two examples of this dual facet. On the one hand, the skin can help us to manage fever, that is, an internal change in temperature because of an ongoing infection, in most cases. We all have experienced the situation of shivering and sweating when we are ill; this is the result of the skin helping the body thermoregulate its own temperature (Kurz, 2008). On the other hand, the skin constantly provides information about the external temperature by activating the sensation of discomfort that we feel when we are too cold or warm so that we are prompt to take actions against these thermal challenges. Thus, interoceptive responses to thermal stimuli can refer both to internally generated stimuli but also in response to the application of thermosensory stimuli on the skin (Muzik & Diwadkar, 2016), both in the cases of heat and cold (Davis et al., 1998; Kwan et al., 2000).

Like the cardiac signals that are always present independently of the extent to which we are aware of them, temperature is perceived constantly via the skin. We are immersed in an external environment characterized by its own temperature, and the skin acts as an interface between the internal functions and the external environment. On a daily basis, we rely on signals mediated by the skin to regulate our homeostatic balance and safety. Failures to regulate body temperature can have dramatic consequences for survival, as well as for physical and cognitive development (for a review, see IJzerman et al., 2015). Because such a narrow window of core body temperature is necessary for optimal functioning, the brain and the body not only rely on bottom-up afferent signals to monitor bodily temperature but also have multiple means of predicting changes in temperature both in and outside the skin to maintain the temperature within the critical range more effectively. Indeed, the interoceptive nature of thermosensation can be investigated not only by focusing on peripheral perception but also by considering descending predictions (for a similar approach in the perception of pain, see Morrison et al., 2013). For example, we do not need to touch an ice cube to know that it is cold: The mere vision of this object provides us with an embodied experience of what it would feel like to touch or be touched by it. Thus, there might be anticipatory processes (see Barrett & Simmons, 2015; Craig et al., 2000; Strigo et al., 2010) taking place at the peripheral and central level that are activated even before any actual threat to thermoneutrality occurs. This process of anticipating thermal status and perceiving temperature generates an affective state of thermal comfort or discomfort, a feeling that signals its homeostatic role and is directly dependent on the body’s needs to seek or avoid certain temperatures (Craig, 2002, 2003; Strigo & Craig, 2016). The feeling of discomfort associated with being hot or cold is the way in which our body communicates that the maintenance of optimal body temperature is key for us to stay alive in changing environmental conditions as “naked apes.” Thus, the skin and thermosensation via the activation of voluntary and involuntary thermoregulatory processes are able to guarantee the maintenance of our interoceptive balance via allostasis (e.g., Burleson & Quigley, 2021; IJzerman et al., 2015). Before turning to the issue of how to quantify thermosensation as interoception in experimental behavioral studies, let us first consider the pathways from the skin to the brain in more detail.

## Specialized Pathways From the Skin to the Brain

A person’s own body, and above all its surface, is a place from which both external and internal perceptions may spring. It is seen like any other object, but to the touch it yields two kinds of sensations, one of which may be equivalent to an internal perception. . . . The self is first and foremost a bodily self.

—Sigmund Freud, “The Ego and the Id”

**Fig. 1.**
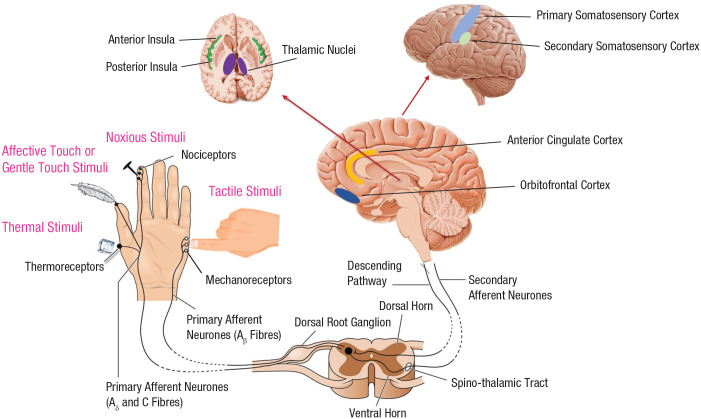
Pathways from the skin to the brain.

The skin is our widest organ in terms of dimension and functions, and it wraps our entire body (Field, 2010; Gallace & Spence, 2014; Montague, 1971; Serino & Haggard, 2010). It is a very sophisticated system both in terms of internal structure and functions, and it is rich in diversity when it comes to specialized peripheral nerves systems activated in response to its stimulation (e.g., Corniani & Saal, 2020). The versatile yet specialized nature of the skin and its afferent systems play an important role in the sense of touch as well as the skin-based interoceptive submodalities under discussion. Broadly speaking, the peripheral receptors in the skin can be mainly classified on the basis of their dimension and conduction velocity. Namely, myelinated fibers (i.e., Aβ) are usually large and provide a fast response to stimulation; in contrast, small fibers provide a relative slower response to stimulation, and they can be unmyelinated fibers (i.e., C) or thinly myelinated (i.e., Aδ). The small, slower fibers are responsible for nociception, thermoception, and affective touch (see Fig. 1). The nociceptors, thermoceptors, and CT receptors are all free nerve endings, the most common nerve ending in the skin, and are sensitive to pressure (very light pressure in the case of CT; extreme pressure in the case of nociceptors), temperatures in different ranges (cold, cool, warm, and hot in the case of thermoceptors; temperature extremes in the case of nociceptors, i.e., > ~40–45 °C or < ~15 °C), or chemicals signaling potential or actual tissue damage (Dubin & Patapoutian, 2010; Jänig, 2018; Olausson et al., 2010). The sensory afferents conveyed by C and Aδ fibers take a distinct pathway during development that reaches the spinal lamina I or solitary tract nucleus, which then connects to the homeostatic/interoceptive nuclei of the thalamus (ventral medial posterior nucleus, or VMPo). In contrast, the faster and larger Aβ fibers connect to the somatosensory/motor thalamic nuclei at a different stage during development (for an overview, see Craig, 2015). Critically, the neural signals carrying information about thermal, nociceptive, and pleasant-touch stimuli from the VMPo then reach the contralateral posterior insular cortex (which is also the target of visceral inputs).

Via this pathway, the spinothalamocortical pathway, the thermal signals not only reach the posterior insular cortex (Craig et al., 2000; Hua et al., 2005) but also activate an autonomous thermoregulatory response in the preoptic area of the hypothalamus (Terrien et al., 2011; Nakamura & Morrison, 2008). From the thalamus and the posterior insula, the thermosensory signals are forwarded to multiple cortical areas, although relatively little is still known about these projections. However, from the posterior insula there are major connections to the anterior insular cortex, where the information is further processed and integrated with other sources of information, including visceral information and exteroceptive signals. The anterior insular cortex has been proposed to assign affective meaning to the information arising from the original thermosensory stimulus (Craig, 2002, 2008; Craig et al., 2000; Diwadkar et al., 2014; Evrard, 2019; Muzik et al., 2020; Satinoff, 1978). In parallel, thermal signals also reach the parietal cortex and the primary somatosensory cortex, possibly subserving sensory discrimination and stimulus localization (Gallace et al., 2014; Solinski & Hoon, 2019), as well as the anterior cingulate cortex and the orbitofrontal cortex (OFC) that might support more motivational dimensions of the thermal experience, including subjective feelings associated with its pleasantness or unpleasantness (Rolls, 2010), which in turn will motivate behavior. The cortical responses associated with thermosensory stimulation have also been described as a hierarchically organized thermoregulatory network that is able to distinguish between cold and warm stimuli (Muzik & Diwadkar, 2016). How the processing of thermoceptive signals turn into affective-interoceptive feelings of thermal comfort and discomfort more exactly is not clear (e.g., Oi et al., 2017), but we speculate that it likely involves an interplay of insular, cingulate, and orbitofrontal areas, and this is an important question for future research.

Gentle-touch stimuli delivered at CT-optimal speed activates the posterior insular cortex in human subjects (Björnsdotter et al., 2009). Activations of the anterior insula, cingulate cortex, and OFC are also seen during affective touch (Case et al., 2016; McGlone et al., 2012; Rolls et al., 2003). Although such gentle stroking also activates the classic somatosensory areas, the primary somatosensory cortex, and the secondary somatosensory cortex (because of the costimulation of larger Aβ fibers), a meta-analysis suggested that the posterior insula is more likely to be activated for affective touch, and primary somatosensory cortices (S1) are more likely to be activated for discriminative touch (Morrison, 2016c). Moreover, pleasantness ratings correlate more than intensity ratings in activity in the cingulate cortex, whereas S1 activity correlated only with intensity ratings, highlighting the cingulate contribution to the affective dimension of the gentle touch (Case et al., 2016). A correlation between neural activity and touch pleasantness has also been reported in the OFC (McCabe et al. 2008), although such correlations are apparently typically not seen for the posterior insula (Case et al., 2016; but see Kress et al., 2011). However, a lesion of the insular cortex after a right-hemisphere stroke disrupts the perception of tactile pleasantness rather than tactile intensity (Kirsch et al., 2020). Thus, pleasantness from gentle touch might arise as a consequence of the further processing of CT signals originating from the posterior insular cortex in the anterior insula, cingulate cortex, and OFC through integration with other sources of information.

Nociceptive processing and the subjective experience of pain are associated with the activation of a network of brain regions, including the primary and secondary somatosensory cortex, the anterior cingulate cortex, and the insular cortices (for a meta-analysis, see, e.g., Duerden & Albanese, 2013; Jensen et al., 2016). Famously, there is no ”primary nociceptive cortex.” Rather, nociceptive signals are processes in several areas, including the insular cortex and the primary somatosensory cortex (area 3a). The subjective experience of pain is thought to arise as a consequence of interactions (Kastrati, Thompson, et al., 2022) between brain regions involved in nociceptive processing (Jensen et al., 2016) and regions supporting cognition and emotion (Geuter et al., 2020). Of particular interest in this context is the processing of nociceptive signals in the midcingulate cortex and bilateral posterior insula (e.g., Perini et al., 2013), which are not only often seen during nociceptive stimulation but also under significant genetic influence (Kastrati, Rosén, et al., 2022), in line with an evolutionally conserved system, which one would expect for a life-sustaining critical interoceptive function.

The similarities in the organization principles of the anatomical pathways and central processing architecture for thermosensation, affective touch, and nociception/pain are one of the key arguments for the proposed redefinition of such modalities as homeostatically relevant and interoceptive because they all carry not only discriminative perceptual qualities but also emotional feelings about the body’s physiological state (Craig, 2003).

## Thermosensation as Skin-Based Interoception: Novel Experimental Directions

Among the skin-based interoceptive submodalities, thermosensation offers numerous advantages from an experimental and methodological point of view. Stimulation can be easily experimentally controlled in the sense that we can systematically manipulate the temperature we deliver on the skin with high precision (e.g., ± 0.1–0.2 °C) while recording the subjective perception (e.g., via a rating scale or detection/discrimination tasks), the objective physiological state of the skin (i.e., temperature), and the physiological reaction (e.g., change in body temperature) to such stimulation (Crucianelli et al., 2022; Radziun et al., 2021). Moreover, it is possible to deliver very selective activation of thermoreceptors in the skin, which can be done with contactless radiant stimulation (heat lamps), dry ice kept at a close distance from the skin, or by laser stimulation. Such stimulation can also be given without an external object touching the skin, which eliminates the potential binding of the thermal experiences to the external object, thus ensuring that the thermal sensations are perceived to be originating from one’s own body. It is also possible to present thermal stimuli that, to various degree, “threatens” thermoneutrality by presenting stimuli that are cooler or warmer than the normal skin temperature to probe the resulting feelings of thermal comfort and discomfort. The latter is an advantage compared with nociceptive and CT-optimal stimulation because feelings of pleasure and pain are “one-directional,” either triggered or absent, rather than changing around a homeostatic target level. Compared with affective touch, it is also easier to selectively activate thermoreceptors than CT afferents (with radiant stimulation or laser). Moreover, in contrast to affective touch or pain, temperature does not necessarily have a strong affective component when manipulated within the innocuous range (cool to warm perception), which is an advantage in experimental studies because it is easier to match conditions and raises fewer ethical issues than when administering pain. Studying interoception via thermosensation is less invasive because it can be prompted externally, unlike other methods used thus far to investigate interoception, such as gastric or bladder functions.

The tasks used to study thermosensation over the last century have focused on the sensory-discriminative nature of this sense in line with most work on somatosensation. We suggest that, by applying the interoceptive principles discussed above, one can design a new generation of thermosensory tasks that more directly probe the affective aspects of thermosensation, such as the subjective feeling of thermal comfort and discomfort.

**Fig. 2.**
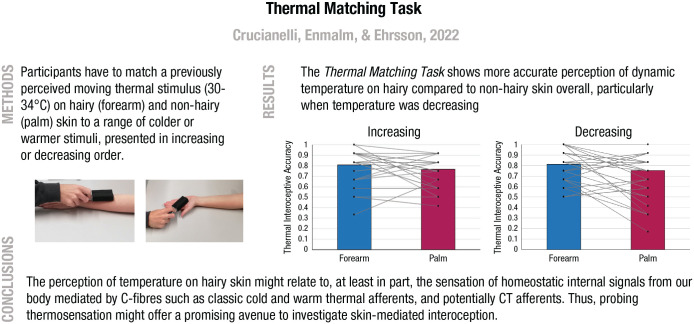
Summary of the methods, results, and conclusions of the thermal matching task, fully described in Crucianelli et al. (2022). In line with classic approaches in the cardiac interoceptive field (e.g., Schandry, 1981), we applied a formula that allowed us to conveniently obtain a number between 0 and 1, where 0 indicates lower ability to detect changes from thermoneutrality (worst performance at the task) and 1 indicates higher such ability (better performance at the task). This index of thermal interoceptive ability can then be compared with similar indexes calculated for other interoceptive tasks in a “battery” of tests to probe different interoceptive submodalities.

In our lab we are currently working on several such new tasks, one of which, the *thermal matching task* (see Fig. 2; Crucianelli et al., 2022; Radziun et al., 2021), is based on concepts from the affective-touch literature and thermosensation as interoception. In this task, participants are asked to recognize a previously perceived moving thermal stimulus applied to the skin at CT-optimal velocity when presented among other warmer or cooler stimuli. The temperatures are within the range of thermoneutrality (30–34 °C), and we register how accurate participants are in detecting thermal stimuli within that range and explore differences between hairy (rich in CT afferents) and nonhairy skin (where CTs are sparse) because CT afferents are tuned to respond optimally to typical skin temperature (Ackerley et al., 2014). The results reveal greater thermosensory sensitivity on hairy skin in line with the idea that temperature perception around thermoneutrality on hairy skin might be based on C fibers such as classic cold and warm thermal afferents (i.e., C and Aδ) and potentially CT signals, which might work in concert with cold and warm receptors by detecting and signaling deviations from their optimal temperature sensitivity (i.e., 32 °C; Björnsdotter et al., 2010; Burleson & Quigley, 2021; Craig, 2009; Morrison, 2016c). This task can be easily extended to include judgments of thermal comfort and the perceived pleasantness of touch and by varying the velocity, temperatures, and skin types stimulated to tease apart the relationships between thermal discrimination, thermal comfort, and tactile pleasantness.

## Skin-Based Interoception in Social Behaviors and Bodily Awareness

Because skin-based interoception provides information about how the skin feels at any given movement, it may also subserve functions in bodily awareness and social interactions. A few studies have suggested a role of CT signals and tactile pleasantness to the sense of the body as one’s own (*body ownership*; Crucianelli et al., 2013, 2018; Lloyd et al., 2013; van Stralen et al., 2014). Body ownership is a multisensory construct whereby different streams of sensory signals are being combined into a coherent multisensory representation of one’s own body (Ehrsson, 2020). Although most previous studies have focused on the integration of visual, tactile, and proprioceptive signals, we know that the sense of body ownership is closely linked to functions of defending the body and emotional-defense reactions (Ehrsson et al., 2007; Graziano & Cooke, 2006), which thus indicate an important role for interoception (Tsakiris, 2017). Ongoing studies have begun to use selective stimulation of thermoreceptors and nociceptors using contactless radial stimulation and laser stimulation to better understand the precise contribution of inputs from thin unmyelinated C fibers to body ownership. Hence, by studying skin-based interoception we can obtain a better understanding about the interplay between exteroception, proprioception, and interoception for the sense of body ownership.

Skin-based interoception supports interpersonal behavioral and social cognition, and probing interoception via thermosensation might offer a particularly intriguing opportunity to study the link between social connection and bodily signals (Arnold et al., 2019). According to some views, the way in which we learn to read, interpret, and respond to thermal signals is also via social tactile interactions with our caregivers (e.g., Ciaunica & Crucianelli, 2019; Ciaunica & Fotopoulou, 2017; Fotopoulou & Tsakiris, 2017). At birth, we do not have the means to act on our interoceptive needs, such as food intake and behavioral thermoregulation (i.e., cover or uncover us up), and we rely on others to take care of our survival. Thus, social touch is a fundamental tool to cope with stressors and challenges via the physiological regulation of our bodily states (Fotopoulou & Tsakiris, 2017; Morrison, 2016c). Social physical contact and proximity such as when hugging and snuggling are also fundamental processes of social thermoregulation, one of the most economical and efficient ways of keeping our body at a good temperature (IJzerman et al., 2015; Morrison, 2016c). Through social-embodied interactions with others, we can guarantee our most optimal social functioning in terms of emotion, thermoregulation, and ultimately survival as a species (for a review, see IJzerman et al., 2015).

## Concluding Remarks

Here, we integrated some of the physiological, behavioral, and neuroanatomical evidence in support of the interoceptive nature of some skin-mediated signals. In particular, we highlighted the strengths and advantages of studying interoception by focusing on the skin because of its dual nature of being exposed to the internal environment of our body and to the external world. We suggested that thermosensation—in addition to affective touch and cutaneous pain—could be considered a valid model of skin-mediated interoception and argued that experimental studies that control for or eliminate the exteroceptive component of thermosensation can allow the interoceptive facet of this modality to be targeted. Moreover, investigating interoception via skin stimulation can provide a unique insight into bodily awareness as well as a better understanding of clinical conditions characterized by disorders of thermoregulation, anhedonia (i.e., the inability to experience pleasure), and chronic pain (i.e., persistent experience of pain past normal healing time), to name a few. Thus, the skin is the sensory organ that can afford us promising opportunities to improve the scientific study and understanding of interoception and its clinical and experimental applications.

## Transparency

*Action Editor:* Laura A. King

*Editor:* Laura A. King
